# Giving a Second Opportunity to Tire Waste: An Alternative Path for the Development of Sustainable Self-Healing Styrene–Butadiene Rubber Compounds Overcoming the Magic Triangle of Tires

**DOI:** 10.3390/polym11122122

**Published:** 2019-12-17

**Authors:** Javier Araujo-Morera, Marianella Hernández Santana, Raquel Verdejo, Miguel Angel López-Manchado

**Affiliations:** Institute of Polymer Science and Technology (ICTP-CSIC), Juan de la Cierva 3, 28006 Madrid, Spain; jaraujo@ictp.csic.es (J.A.-M.); rverdejo@ictp.csic.es (R.V.); lmanchado@ictp.csic.es (M.A.L.-M.)

**Keywords:** styrene–butadiene rubber, sustainability, self-healing, ground tire rubber, magic triangle, molecular dynamics

## Abstract

Current regulations demand tires with long lifetime and reduced fuel consumption without sacrificing car safety. However, tire technology still needs to reach a suitable balance between these three indicators. Here, we address them by developing a self-healing tire compound using styrene–butadiene rubber (SBR) as the matrix and reclaimed tire waste as the sustainable filler. The addition of ground tire rubber (GTR) to the matrix simultaneously improved the rolling resistance and maintained both wet grip and healing ability. We provide an in-depth analysis of the healing behavior of the material at a scale close to the relevant molecular processes through a systematic dynamic-mechanical and dielectric analysis. We found that SBR and SBR/GTR compounds show a complete recovery of stiffness and relaxation dynamics after being damaged by cyclic deformation, resulting in a heterogeneous repaired rubber network. This new development could well overcome the so-called magic triangle of tires, which is certainly one of the key objectives of the tire industry.

## 1. Introduction

One of the most serious environmental problems facing society today is the accumulation of a great quantity of used tires in landfill sites, and the increasing automotive production over the years is continuously aggravating it [1]. Recycling and recovering used tires can be considered reasonable strategies to solve this concern. However, some limitations have to be defeated, since a tire is composed of various materials and additives incorporated in the rubber compound, turning it into a very complex system [2,3]. The more stringent environmental regulations and the fluctuating price of raw materials are the driving forces for the development of several innovative recycling technologies [4]. The recycling of waste tires has important implications in energy conservation, environmental protection, costs reduction, and in promoting the “4R” principle (Re-use, Reduce, Recycle, and Recover) [2,5,6]. One of the main ways to consume tire waste is in the production of technically less demanding rubbers by mixing rubber powder (i.e., grinding the rubber waste to form granulates) with new rubbers (or plastics) [7]. Sports and playing surfaces, floors and walkway tiles, concrete mixtures [8], asphalt mixtures [9], thermal and acoustic isolation, and footwear, among others, are traditional fields of application of ground tire rubber (GTR).

More recently, dedicated efforts have been put forth to convert GTR into other added value products with improved performance [1]. Research concerning the incorporation of GTR in natural rubber (NR) demonstrated that good tensile strength and abrasion resistance were maintained, especially when using up to 10 parts per hundred rubber (phr) of smaller size GTR [10]. Furthermore, the use of 20 % of GTR has been shown to improve the mechanical performance of butadiene rubber (BR) and styrene–butadiene rubber (SBR) [11].

Another recent application of GTR is as a potential filler in self-healing matrices [12]. Self-healing materials are designed with the intention of extending their life span, by fully or partially repairing damage autonomously, without any external stimulus. Several healing concepts have been studied and applied to rubber matrices [13,14,15,16,17,18]. The irreversible covalent crosslinked rubber network theoretically blocks its self-healing capability, since chain mobility is required for healing to take place. Consequently, the mechanical performance and healing efficiency are antagonistic properties, since the simultaneous improvement of both properties is a priori not achievable [19]. Overcoming this compromise is one of the actual challenges of the self-healing scientific community. Previously [12], the authors reported that the addition of 10 phr of GTR to a SBR matrix allows prevailing over such compromise, since tensile strength increased by 80 % and healing efficiency was maintained at 50 %. Meanwhile, the equivalent compound with 10 phr of a typical filler, such as carbon black, reinforced the matrix but hindered the healing ability of the compound [12].

Many elastomeric products are used in vehicles under dynamic conditions. Tires, beltings, and engine mountings are subject to rapid repeated deformations. Therefore, a considerable number of investigations have been carried out using dynamic mechanical analysis (DMA) to study these dynamic properties over a wide range of strain amplitudes. This methodology provides important information on rubber characteristics, such as the glass transition temperature (T_g_) and frequency–temperature characteristics that are expressed in terms of master curves. These curves characterize the molecular motions of the polymeric chains [20]. The molecular dynamics of elastomeric systems can also be studied using broadband dielectric spectroscopy (BDS). This powerful and sensitive tool for studying the dipolar relaxations of polymers offers the possibility of covering an extensive range of temperatures and frequencies. Therefore, it is possible to investigate dynamic processes that take place on different time scales [19,21]. BDS can also be considered an important practice for monitoring healing in polymers, considering that this technique is capable of analyzing and describing multilevel molecular mobility as a function of the structural state of the polymer [19,22]. Hernández Santana et al. studied, for the first time, the molecular dynamics evolution during the healing of both internal and macroscopic damages of a partially cured NR, demonstrating that the polymer structure in the different states (virgin and healed) diverges [19]. More recently, Patnaik et al. used BDS for unraveling the mechanism of the healing process in NR–silica composites [23]. Analyzing the interrelation between dynamics and molecular structure serves to elucidate the physical properties and the possible repair mechanism. Therefore, the interpretation of the molecular mobility in terms of DMA and BDS is of high practical interest.

Herein, we have performed a systematic dynamic mechanical and dielectric analysis in an attempt to examine the healing of SBR/GTR compounds at a scale close to the relevant molecular processes and the state of the material at the end of the healing treatment. The rubber compounds were investigated by both methods under three different conditions—virgin, damaged under cyclic deformation and repaired—to comprehend the effect of GTR content on the relaxation mechanisms and healing capability of the rubber matrix. We also analyzed the effect of the GTR content on the three major indices of the magic triangle of tires (fuel economy, driving safety, and lifetime). The results obtained will be advantageous for understanding the performance of sustainable rubber compounds in a dynamic, variable frequency environment, with potential application for the tire industry and as alternative strategy for the management of end-of-life vehicles.

## 2. Materials and Methods

### 2.1. Materials and Compounding

Styrene–butadiene rubber (SBR) (BUNA SE1502H, Arlanxeo, Maastricht, The Netherlands) and commercial grade vulcanizing additives were used as received. bis [3-(trietoxysilyl)propyl]tetrasulfide (TESPT, Evonik Industries AG, Essen, Germany) was used as the coupling agent. Ground tire rubber (GTR) obtained by ambient grinding used tires (combination of passenger car and truck tires of unknown composition) was supplied by Signus Ecovalor (Madrid, Spain). The previously reported characterization of the as-received GTR showed an average composition of 55 phr NR, 45 phr SBR/BR, and 50 phr of carbon black (CB). The full characterization of as-received GTR has been reported elsewhere [12]. As-received GTR was further pulverized under cryogenic conditions using a Cryomill (Retsch, Haan, Germany), following an optimized protocol consisting of 18 cycles with the sequence of time and frequency schematically presented in Figure S1-1 (Supplementary Information S1). Rubber compounds were prepared according to the compositions shown in Table 1.

The mixing was carried out in an open two-roll mill (MGN-300S, Comerio Ercole, Busto Arsizio VA, Italy) using a rotor speed ratio of 1:1.5 at room temperature. First, rubber was passed through the rolls until a band was formed. The activating complexes, zinc oxide (ZnO) and stearic acid (SA), and curatives, N-cyclohexylbenzothiazole-2-sulphenamide (CBS) and sulfur (S), (Sigma Aldrich, St. Louis, MO, USA) were sequentially added to the rubber. In the case of the filled compounds, a pre-mix of SBR, GTR, and TESPT was prepared in an internal mixer (Haake™ Rheomix 600, Thermo Fisher Scientific, Waltham MA, USA) at 100 °C and a rotor speed of 100 rpm during 7 min. The pre-mixed material was incorporated with the rest of ingredients in the two-roll mill for further mixing and improving the processability of the rubber compound, following the mixing protocol previously described [24].

The crosslinking process was monitored checking the torque variation as function of time using a Rubber Process Analyzer (RPA2000, Alpha Technologies, Akron, OH, USA) at a frequency of 0.833 Hz, 2.79 % strain for 60 min at curing temperature T_c_ = 160 °C. Then, the compounds were vulcanized in an electrically heated hydraulic press (Gumix, Barcelona, Spain) at 160 °C and 20 MPa according to their t_97_ derived from the curing curves data. Samples were cut out from press-cured sheets to perform damage and healing tests. Pure GTR sheets were also prepared in the hydraulic press at 160 °C during 10 min [25].

### 2.2. Material Characterization

#### 2.2.1. X-ray Photoelectron Spectroscopy (XPS)

X-ray photoelectron measurements were performed on the surface of as-received GTR and cryo grounded GTR using a spectrometer (MT500, Fisons, Loughborough, UK) equipped with a hemispherical electron analyzer (CLAM 2) and a Mg Ka X-ray source (1253.6 eV) operated at 300 W. Binding energies were corrected to the carbon 1s peak located at 285 eV.

#### 2.2.2. Cyclic Deformation Testing

All compounds were subjected to a cyclic deformation protocol. Tests were done on a universal mechanical testing machine (Instron3366, Grand Rapids, MI, USA) equipped with a 1 kN load cell at room temperature. Rectangular samples (50 mm × 4 mm × 2 mm) and films (50 mm × 25 mm × 0.3 mm) were tested during 20 stretching cycles at a speed of 500 mm/min up to 70 % of the maximum deformation of each compound.

#### 2.2.3. Healing Protocol

After being damaged by cyclic deformation, the samples were kept for 12 h at room temperature and then placed in an oven (Thermo Scientific, Waltham, MA, USA) for 12 h at 70 °C. After applying this thermal healing treatment, the samples were retested following the same cyclic test conditions. Healing efficiency (η) was quantified as follows:

η[%] = (P_healed_)_/_(P_virgin_) × 100 (1)

where P_healed_ and P_virgin_ are the values of a selected property in the healed and virgin state, respectively.

Macroscopically damaged (fractured) samples by tensile tests were also healed following the healing protocol described above. Healing efficiency based on the recovery of tensile strength is reported in Table S2-1 (Supplementary Information S2).

#### 2.2.4. Dynamic Properties

Virgin, damaged (by cyclic deformation), and repaired compounds, as well as GTR samples, were characterized using dynamic mechanical analysis (DMA) and broadband dielectric spectroscopy (BDS). DMA spectra were registered using a DMA Q800 device (TA Instruments, New Castle, DE, USA). Dog-bone specimens were tested in tensile mode at a static preload of 0.1 N with a superimposed sinusoidal 0.2 % strain. The spectra were taken in a temperature range of −100 to 100 °C at a frequency of 50 Hz. The temperature ramp was 2 °C/min. A high-resolution dielectric analyzer ALPHA (Novocontrol Technologies Gmbh, Montabaur, Germany) was used for BDS measurements. The films were placed in the dielectric cell between two 20-mm diameter parallel gold electrodes. Frequency sweeps between 10^−1^ and 10^6^ Hz were performed from −100 to 100 °C with a step of 5 °C.

## 3. Results

### 3.1. Effect of GTR Content on the Dynamic Properties of Pristine SBR Compounds

#### 3.1.1. Dynamic-Mechanical Analysis

**Figure 1:**
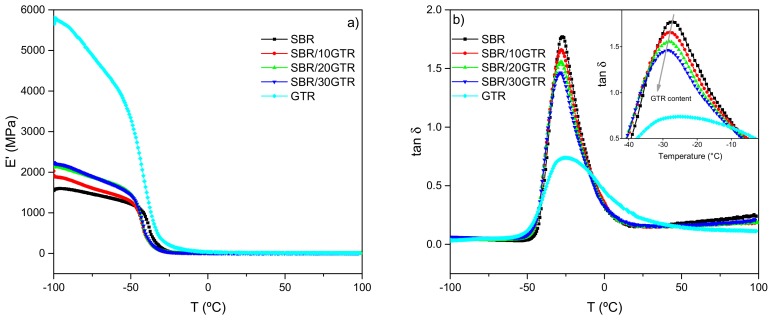
(**a**) Storage modulus (E′); (**b**) Damping factor (tanδ) of SBR/GTR compounds.

The temperature dependence of the storage modulus (E′) and damping factor (tan δ) at a fixed frequency is a good measuring tool to evaluate the effect of the underlying structural properties, such as the crosslinking degree and rubber–filler interactions, on the mechanical performance of a rubber matrix. As it can be seen in Figure 1, GTR presents the maximum E′ associated with the high content of carbon black in its composition, SBR has the lowest values, and the SBR/GTR compounds exhibit intermediate values. The observed increase in E′ for the SBR/GTR compounds with respect to the unfilled SBR matrix can be attributed to two factors. First, carbon black within GTR restricts the mobility of the rubber chains, providing rigidity to the vulcanized compounds [5,26,27]. Secondly, chain mobility also decreases due to the increase in the crosslink density of the vulcanizates in the presence of GTR (see Table S2-1, Supplementary Information S2). This notorious increase in crosslink density, especially with 10 phr of GTR, could be demonstrating a more intensive vulcanization process due to the presence of TESPT [12] and of additional sulfur released during the cryo-grinding process of GTR [28]. This additional sulfur is observed on the S2p core spectrum of the as-received GTR and cryo-grounded GTR from XPS measurements (see Figure S2-2, Supplementary Information S2). The S2p spectra were deconvoluted into S–C (162.3 eV), S–S (164 eV), and S–O (165.7 eV) components. After the cryo-grinding step, large decreases were observed on the S–S peak (64 %) and S–C peak (40 %) areas, and at the same time, the intensity of the oxidized sulfur groups (S–O) increased. These results allow us to infer that the crosslinked sulfur bonds were partially destroyed, i.e., there was a partial devulcanization process. The cryogenic conditions promote the formation of free sulfur radicals, and enable active sulfur to be converted to sulfate and/or oxidation products [29].

Figure 1b shows the dependence of the loss factor (tan δ) with temperature. As expected, the filled compounds have an intermediate behavior between GTR and SBR, which show the minimum and maximum value of tan δ, respectively. The loading effects at different temperature regions are governed by different mechanisms. At temperatures near the tan δ peak (transition zone), two opposite effects are to be considered. First, the presence of carbon black in GTR gives a low hysteresis for a specific energy input, since individual filler particles may not absorb energy significantly [30]. Secondly, in this transition zone, the total rubber fraction in the compound is responsible for the high portion of energy dissipation among rubber molecules, resulting in high hysteresis. For this set of compounds, the governing factor seems to be the carbon black content, since the value of tan δ decreases as the GTR loading increases. Moreover, the peak temperature (i.e., glass transition temperature) slightly shifts to lower temperatures as the GTR content increases due to the presence of NR, which has a T_g_ around −60 °C. The GTR behavior also shows a broader peak evidencing the contribution of both SBR and NR contained in GTR.

DMA measurements are also of the utmost interest for the tire industry, since the prediction of tire performance, e.g., fuel saving efficiency and driving safety, is usually evaluated by considering the viscoelastic properties of rubber. Tan δ is frequently used to foresee the rolling resistance and the wet grip of tires, these two parameters being linked to fuel economy and driving safety, respectively [31]. The basic theory behind these parameters is related to the hysteresis of the rubber compound by the continuous deformation of the sample at different temperatures. During driving, tires rotate at relatively low frequencies (~10^1^–10^2^ Hz), whereas the temperature reached by the tire tread is ~60–70 °C due to the energy dissipation of rubber during the dynamic deformation. In general, the lower tan δ value at 60 °C indicates lower rolling resistance, and thus higher fuel-saving efficiency. On the other hand, when full brakes are applied during driving on a wet road, the tire tread will be subjected to periodical deformation at very high frequencies (~10^4^–10^5^ Hz) due to the dynamic sliding contact on the road roughness. Based on the Williams–Landel–Ferry (WLF) equation, this high frequency could be estimated by shifting the measured temperature toward lower temperatures. Hence, the wet grip efficiency of rubber can be predicted considering the tan δ value at −10–0 °C; higher values imply better wet grip [26,31,32,33]. Then, the ideal condition would be to achieve a high tan δ at −10 °C to assure appropriate wet traction; simultaneously, a low tan δ at 60 °C would guarantee lower rolling resistance and better fuel economy.

**Figure 2:**
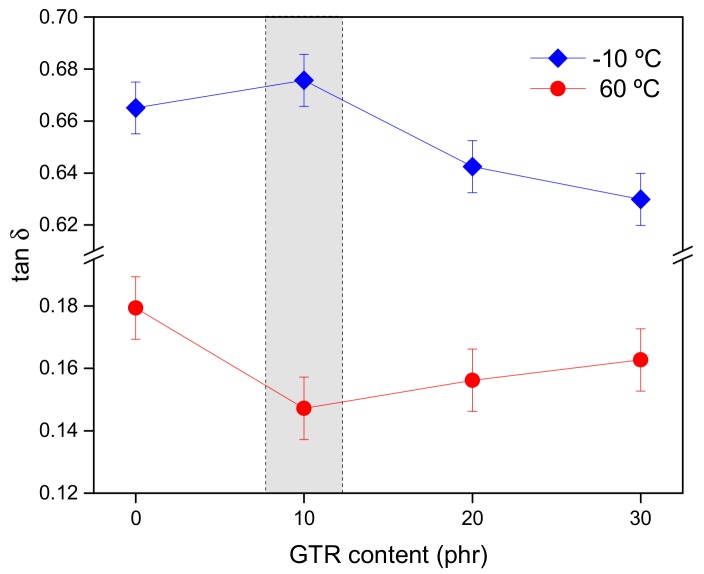
Damping factor (tan δ) of SBR/GTR compounds at low (−10 °C) and high (60 °C) temperatures.

As seen in Figure 2, the SBR/10GTR compound has the overall best performance, since the wet grip and rolling resistance are their maximum and minimum, respectively. These results suggest that small amounts of GTR (10 phr) are well dispersed in the SBR matrix enhancing the rubber–filler interactions [34]. Similar results were found by Kim et al. [35]. They studied SBR/NR blends with a bio-based epoxidized soybean oil and with enhanced interaction with silica resulting in improved wet grip and low rolling resistance. Fracture surfaces of SBR/GTR compounds are presented in Figure S3-1 (Supplementary Information S3). The SBR/10GTR compound shows a good dispersion of GTR in the matrix, reflecting a smooth surface without voids. With the increment of GTR content (20 and 30 phr), a more heterogeneous and rough surface can be seen, and some agglomerates appear. Moreover, in terms of tread composition, low rolling resistance is favored by compounds of lower hysteresis. Hence, the presence of NR (which is a very low hysteresis rubber) in the GTR composition could also be a key factor improving the performance in terms of rolling resistance [36]. Nonetheless, at higher GTR loading (20 and 30 phr), the formation of agglomerated structures seems to dominate versus the increased amount of NR, leading to higher values of tan δ at 60 °C, and hence a higher rolling resistance [34]. Other authors have also found that the filler dispersion is the dominant factor governing the rolling resistance of vulcanizates [37]. As for the wet grip, the addition of a higher amount of GTR also implies a higher amount of carbon black, forming agglomerates that reduce the flexibility needed at low temperatures. Ren et al. [24] found similar behavior on SBR compounds filled with carbon black and fly ash. The results herein reported represent a novel and added value to the use of GTR as filler in SBR compounds, since it does not deteriorate the aforementioned requirements of fuel economy and driving safety relevant to the tire tread.

#### 3.1.2. Broadband Dielectric Spectroscopy (BDS) Analysis

**Figure 3:**
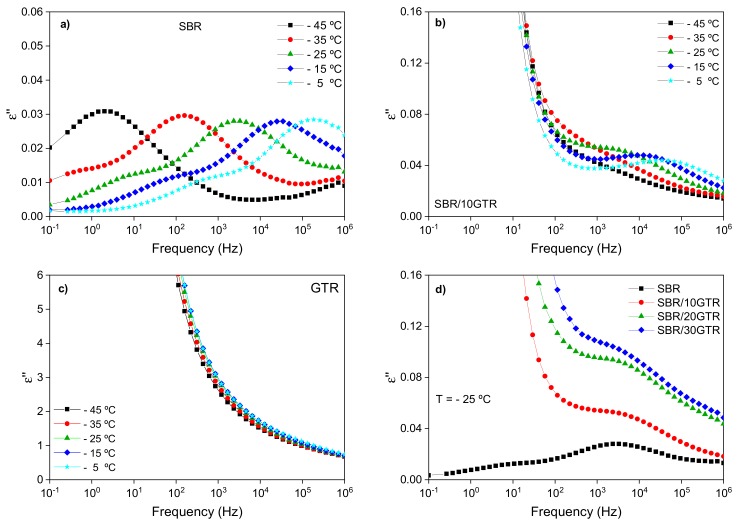
Dielectric loss (ε″) as a function of frequency in the temperature range from −45 to −5 °C of: (**a**) SBR compound; (**b**) SBR/10 GTR compound, and (**c**) GTR. (**d**) Dielectric loss (ε″) as a function of GTR content at a selected temperature T= −25 °C.

Figure 3a shows the dielectric loss (ε″) spectra as a function of frequency for unfilled SBR in a temperature range near its glass transition temperature (−50 °C). A maximum corresponding to the relaxation of SBR chain segments is observed, which evolves toward higher frequencies as the temperature increases. Figure 3b shows the dielectric loss ε″ of a SBR compound filled with 10 phr of GTR. Both 20 and 30 phr GTR filled compounds show equal behavior and are reported in Figure S4-1 (Supplementary Information S4). The same segmental relaxation is observed as in the unfilled compound. However, it presents a clear difference at low frequencies. The significant rise in ε″ is a consequence of the conductivity associated with the carbon black present in the GTR (Figure 3c). In addition, the contribution of conductivity is more noticeable as the content of GTR increases, masking almost completely the SBR relaxation, as seen in Figure 3d [26].

The dependence of the electrical conductivity with frequency is shown in Figure S4-2 (Supplementary Information S4). The filled compounds show two clearly differentiated zones: one at frequencies <10^2^ Hz, where the conductivity remains constant and increases with the GTR content, and another at frequencies >10^2^ Hz, where a linear dependence with frequency is observed. This behavior is typical of composite materials and is described by the percolation theory. One can observe that incorporating GTR increases the conductivity of the material by three orders of magnitude, from 10^−15^ to 10^−12^ S/cm on average, caused by the creation of an interconnected percolative filler network in the rubber matrix [38,39,40].

### 3.2. Healing Performance of SBR/GTR Compounds

#### 3.2.1. Correlating Dynamics and Structure with Healing

In order to elucidate the physical properties of the developed compounds and their possible repair mechanism after cyclic deformation damage, we have analyzed the interrelation between dynamics and molecular structure by means of DMA and BDS tests.

**Figure 4:**
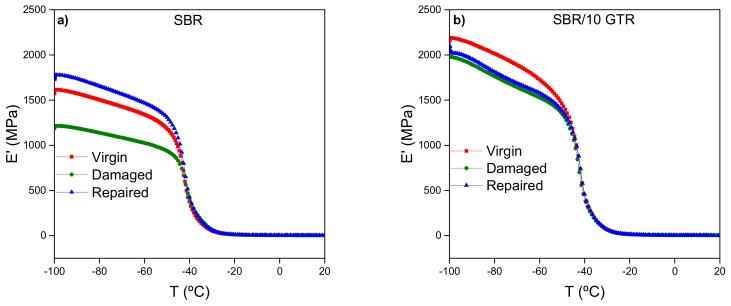
Storage modulus (E′) as a function of temperature for: (**a**) SBR and (**b**) SBR/10GTR compound.

Figure 4 shows E′ as a function of temperature for SBR and SBR/10GTR compounds under three different test conditions (virgin, damaged, and repaired). A decrease in E′ is observed for the damaged samples. Such a behavior is attributed to the cyclic deformation to which the material was subjected and can be interpreted based on the Mullins effect. This effect is characterized by a softening induced by the deformation of an elastomer after a first load during a protocol of several load cycles [41]. The main physical interpretations that explain the Mullins effect are (i) the breaking of bonds (e.g., sulfur crosslinks) or chain scission of rubber molecules, (ii) the physical disentanglement of rubber chains, (iii) the breakage of filler aggregates, and (iv) the consideration that the elastomeric material is constituted by a soft phase and a rigid one (groups of molecular chains joined by segments of short-chain entanglements or intermolecular forces), being the latter broken and transformed into soft regions during deformation [40,41,42]. Consequently, for the unfilled SBR compound, the decrease in E′ could mainly be due to the broken chains transformed into dangling chains, which do not contribute to the material stiffness [41]. Instead, the decrease in E′ for the SBR/GTR compound can be a consequence of the disentanglement of chains and breakage of the rubber network, together with the fracture of filler aggregates and/or rupture of the carbon black network [43].

After the thermal healing protocol application, an increase and thus recovery of E′ is observed. This behavior is accentuated for the unfilled SBR compound, resulting in an even higher E′ value than that of the virgin material (η=110%). Recovery caused by heating could be explained by the arrangement of a new network at sufficiently high temperature thanks to the formation of entanglements between dangling chains [44]. Similar behavior was reported by Patnaik et al. in NR [23]. They ascribed the redistribution of the structure to the formation of a new network with different architecture and higher modulus.

Several authors [45,46] have established that for intrinsic macroscopic healing to occur, three main steps should be followed: firstly, a self-adhesion step (interface formation); secondly, a long-range chain diffusion at short time scales (interphase formation); and finally, a homogenization process at longer times (randomization). The full randomization only occurs if fracture does not reappear at the same site of the initial damage. Thus, a valid explanation to the total recovery of stiffness in unfilled SBR could be the interdiffusion of the dangling chains, which was promoted by the reversibility of the disulfide and polysulfide bonds of the vulcanized network, reestablishing the chemical bonds through the damaged network [16,47,48].

In the case of the SBR/10GTR compound, there is an almost complete recovery of stiffness with temperature (η=96%), which suggests that heating the samples increases the molecular mobility and free chains adsorbed at the CB aggregates surface, creating stronger rubber–filler interactions. New entanglements and the layer of bonded rubber at the rubber–filler interface are believed to play a key role, resulting in the recovery of the stiffness, and hence the healing of the SBR/10GTR compound [43,44].

**Figure 5:**
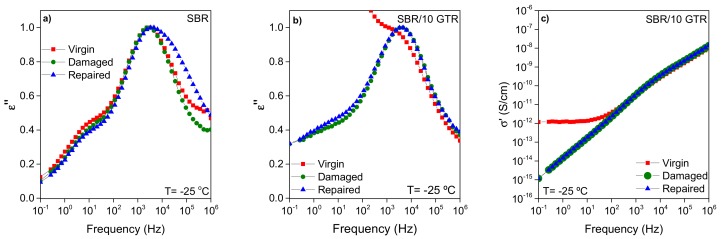
Dielectric magnitudes (ε″ and σ′) as a function of the frequency at a temperature of −25 °C for the virgin, damaged, and repaired states of: (**a**) SBR; (**b**,**c**) SBR/10GTR compound.

BDS gave some additional insights into the dynamics and polymer architecture of the rubber compounds under the three different test conditions. By plotting the normalized dielectric loss in a broad frequency range, one can discriminate how the shape (symmetry, broadness) of the relaxation spectra varies. Figure 5 shows the normalized plots for unfilled SBR and that filled with 10 phr of GTR at a selected temperature (T = −25 °C). At this frequency window, the dielectric relaxation appears as a well-resolved peak. The data corresponding to the virgin and damaged state of SBR (Figure 5a) do not differ between each other; the frequency where the maxima appear are the same, and the shape of the peak is similar. This could imply that the damaged produced in the network structure did not alter the overall relaxation dynamics of the rubber, although we were expecting less restricted dynamics due to the bond breakage and chain scissions occurring after cyclic deformation [19]. Nonetheless, clear differences in the shape of the spectra can be noticed in the repaired sample; a widening of the relaxation is observed. The phenomenological model of Schönhals and Schlosser [49] proposes that the shape of the dielectric loss peak is related to the behavior of the polymer at low and high frequencies, controlled by inter- and intra-molecular interactions, respectively. The application of such a model to the studied SBR suggests that the variations on the high-frequency side in the healed sample can be attributed to new entanglements that are formed thanks to local chain mobility/diffusion favored by the thermal healing treatment [23,50]. This means chain segments with different dynamics and thus a higher degree of structural heterogeneity, implying regions of distinct mobility. This is in accordance with the DMA results, where the new entanglements formed are responsible for the recovery and increase in E′.

In the case of the compound filled with 10 phr of GTR (Figure 5b), a significant change is observed between the virgin, damaged, and repaired states. Upon damage, the percolation path is lost, and the notorious rise in ε″ at low frequencies ascribed to the carbon black present in the GTR is not seen. Therefore, the softening of the compound after damage and the drop of conductivity can be correlated and associated to the carbon black structure and conductive network breakdown, as seen in Figure 5c [51,52]. Once the repair process has taken place, the material conductivity remains low, indicating that the alteration of the filler network is not recovered [43]. It seems that the repair protocol used in this research was not effective enough to form again the contact and alignment of the conductive particles. Further work will consider different healing protocols in order to restore the structural properties as well as electrical conduction.

#### 3.2.2. Healing as Part of the Magic Triangle of Tires

The “magic triangle” principle of the tire industry is related to three main material-specific requirements relevant to the tread, such as rolling resistance, wet grip, and abrasion resistance; targeting lower fuel consumption, driving safety, and extended lifetime, respectively. Instead of studying abrasion resistance, in this research, we have redefined the extended lifetime requirement by conferring healing ability to the rubber compounds after being exposed to cyclic deformation damage.

**Figure 6:**
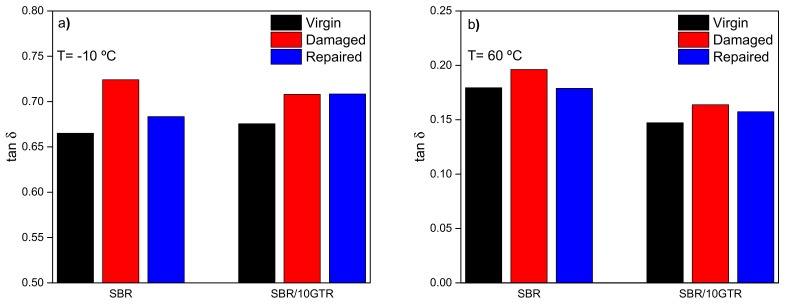
Damping factor (tan δ) of SBR and SBR/10GTR compounds under virgin, damaged, and repaired conditions at: (**a**) low temperatures (−10 °C); (**b**) elevated temperatures (60 °C).

SBR and SBR/10GTR compounds were studied in order to analyze the healing protocol effect on the viscoelastic properties associated with the final performance of the tires (wet grip and rolling resistance). Figure 6 presents the values of tan δ at low (−10 °C) and at high (60 °C) temperatures. In the damaged state, tan δ tends to increase at both temperatures for both the SBR and SBR/GTR compounds. At low temperatures, a higher energy dissipation due to the increased rubber chains slippage and to the disruption of the filler network results in high values of tan δ [37]. While at 60 °C, the loss of conformational entropy with the cyclic deformation, the disentanglement of network chains, and the destruction of the filler network are reflected in a higher value of tan δ [34]. After the healing protocol is applied, tan δ at both temperatures lowers down, achieving an almost complete recovery of the initial value. This behavior corroborates the creation of a new network with entanglements and enhanced rubber–filler interactions [44].

**Figure 7:**
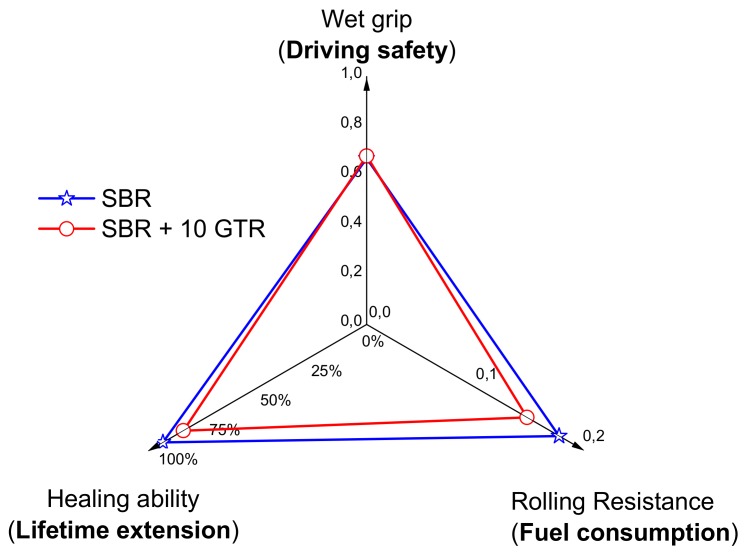
Rolling resistance, wet grip, and healing ability of SBR and SBR/10GTR compounds.

Finally, the representation of the magic triangle, as seen in Figure 7, clearly summarizes the results. The addition of GTR is an effective way to lower fuel consumption, maintain wet grip, and allow an extended lifetime thanks to a full recovery of the stiffness and dynamic properties after being exposed to cyclic deformation damage.

## 4. Conclusions

In the present work, we attempt to comprehend the changes in structure and dynamics related to SBR compounds filled with GTR under three different conditions: virgin, damaged by cyclic deformation, and thermally healed. The interpretation of healing in terms of dynamic properties is of high practical interest, since most rubber products are used in a frequency-dependent environment. Based on a series of systematic and complementary experiments by DMA and BDS, we show that SBR/GTR compounds fully recover their stiffness and relaxation times; meanwhile, the structure of the healed rubber network becomes more heterogeneous, suggesting chain interdiffusion and the reversibility of the disulfide bonds correlated to the healing process.

We also customize the magic triangle of tires, substituting abrasion resistance for healing ability. The presence of a sustainable filler, such as GTR, optimizes the three triangle indices overcoming the delicate balance between rolling resistance, wet grip, and healing efficiency. In conclusion, the material herein studied can be useful for developing new applications that are economically and environmentally convenient for the tire industry, combining healability and sustainability features together.

## Figures and Tables

**Table 1:** Styrene–butadiene rubber (SBR) compound recipes. CBS: N-cyclohexylbenzothiazole-2-sulphenamide, GTR: ground tire rubber, S: sulfur, SA: stearic acid, TESPT: bis [3-(trietoxysilyl)propyl]tetrasulfide.

Ingredients(phr)	Compound			
	SBR	SBR/10GTR	SBR/20GTR	SBR/30GTR
SBR	100	100	100	100
ZnO	5	5	5	5
SA	1	1	1	1
CBS	0.70	0.70	0.70	0.70
S	0.70	0.70	0.70	0.70
TESPT	0	5	5	5
Cryo-grounded GTR	0	10	20	30

## Supplementary Materials

The following are available online at https://www.mdpi.com/2073-4360/11/12/2122/s1, Figure S1-1: Schematic representation of grinding and cooling cycles. Figure S2-1: Stress–strain curves of the SBR/GTR compounds in virgin (V) and repaired (R) state. Table S2-1: Mechanical properties of pristine and repaired SBR compounds. Figure S2-2: The S2p core spectrum of as received GTR and cryo grounded GTR. Figure S3-1: Scanning electron microscope (SEM) images of fracture surface of SBR/GTR compounds. Figure S4-1: Dielectric loss (ε″) as a function of the frequency of: (a) SBR/20GTR; (b) SBR/30GTR, in the temperature range from −45 to −5 °C. Figure S4-2: Electrical conductivity (σ′) as a function of frequency of SBR/GTR compounds at 25 °C.
