# Supplementary material for: Giving a Second Opportunity to Tire Waste: An Alternative Path for the Development of Sustainable Self-Healing Styrene–Butadiene Rubber Compounds Overcoming the Magic Triangle of Tires
Source: Polymers (Basel). 2019 Dec 17;11(12):2122. doi: 10.3390/polym11122122 (PMC6960816; doi:10.3390/polym11122122)
Supplement: Supplementary file 1 [file polymers-11-02122-s001.pdf]

SUPPLEMENTARY MATERIALS

Giving a second opportunity to tire waste: an alternative path for the development of sustainable self-healing styrene-butadiene rubber compounds overcoming the magic triangle of tires

Javier Araujo-Morera; Marianella Hernández Santana*; Raquel Verdejo; Miguel Angel López-Manchado

Institute of Polymer Science and Technology (ICTP-CSIC), Juan de la Cierva 3, Madrid 28006, Spain. jaraujo@ictp.csic.es (J.A.M.); marherna@ictp.csic.es (M.H.S.); rverdejo@ictp.csic.es (R.V.); lmanchado@ictp.csic.es (M.L.M.)

* Correspondence: marherna@ictp.csic.es (M. H. S.)

S1. Cryo-grinding protocol

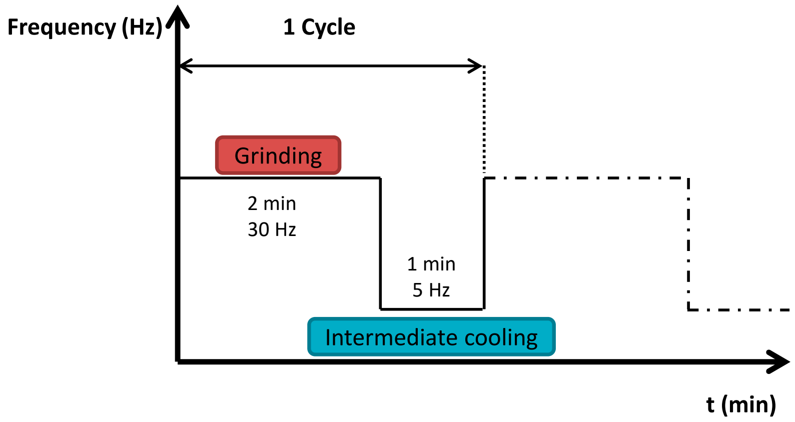

Figure S1-1. Schematic representation of grinding and cooling cycles.

S2. Tensile curves of virgin and repaired SBR/GTR compounds

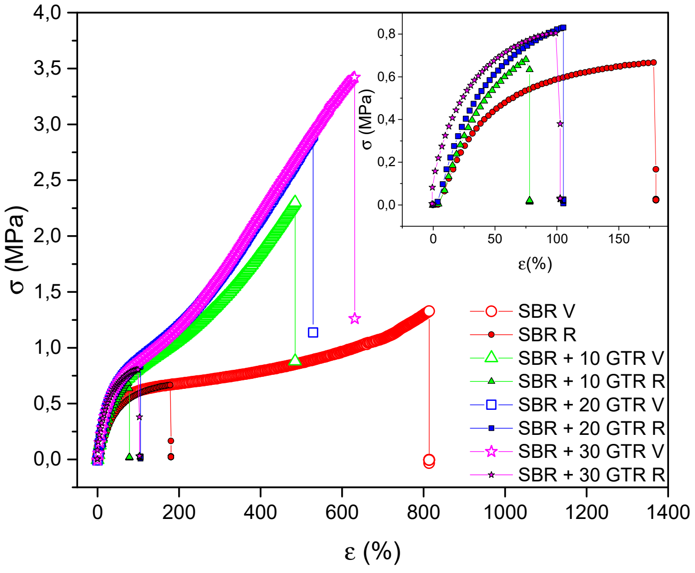

Figure S2-1. Stress-strain curves of the SBR/GTR compounds in virgin (V) and repaired (R) state.

Table S2-1. Mechanical properties of pristine and repaired SBR compounds.

	**Compound**				
	**SBR**	**GTR**	**SBR/10GTR**	**SBR/20GTR**	**SBR/30GTR**
PRISTINE					
Tensile stress at 50% strain, σ_{50} (MPa)	0.54±0.02	1.27±0.05	0.65±0.02	0.66±0.02	0.59±0.04
Tensile stress at 100% strain, σ_{100} (MPa)	0.65±0.02	2.25±0.05	0.83±0.03	0.86±0.03	0.77±0.04
Tensile stress at 300% strain, σ_{300} (MPa)	0.75±0.02	-	1.42±0.06	1.64±0.07	1.58±0.03
Tensile stress at 500% strain, σ_{500} (MPa)	0.88±0.02	-	2.40±0.10	2.79±0.09	2.61±0.07
Tensile strength, σ_b (MPa)	1.33±0.08	4.8±0.2	2.60±0.20	2.90±0.10	3.30±0.10
Elongation at break, ε_b (%)	846±34	198±9	550±24	546±18	639±24
Crosslink density, $\nu \times 10^{-5}$ (mol/g)	1.46±0.02	30.1±0.3	4.83±0.06	4.35±0.08	3.24±0.04
REPAIRED					
Tensile stress at 50% strain, σ_{50} (MPa)	0.54±0.04	-	0.68±0.02	0.65±0.03	0.66±0.01
Tensile stress at 100% strain, σ_{100} (MPa)	0.67±0.05	-	0.86±0.03	0.85±0.03	0.83±0.01
Tensile stress at 300% strain, σ_{300} (MPa)	-	-	-	-	-
Tensile stress at 500% strain, σ_{500} (MPa)	-	-	-	-	-
Tensile strength, σ_b (MPa)	0.75±0.05	-	0.80±0.10	0.97±0.08	0.91±0.02
Elongation at break, ε_b (%)	177±14	-	98±9	180±55	228±66
Healing efficiency, η (%)	56±7	-	31±5	33±3	28±1

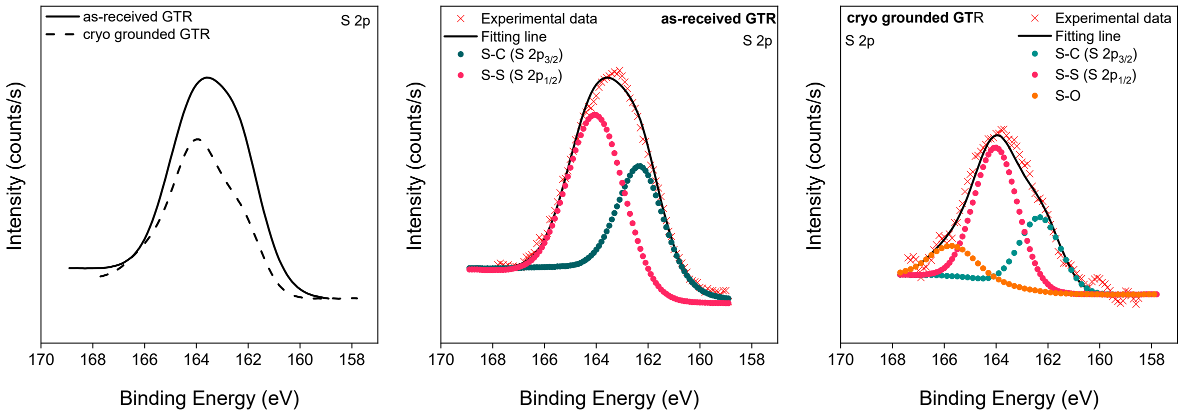

Figure S2-2. The S2p core spectrum of as received GTR and cryo grounded GTR.

S3. Fracture surface of SBR/GTR compounds.

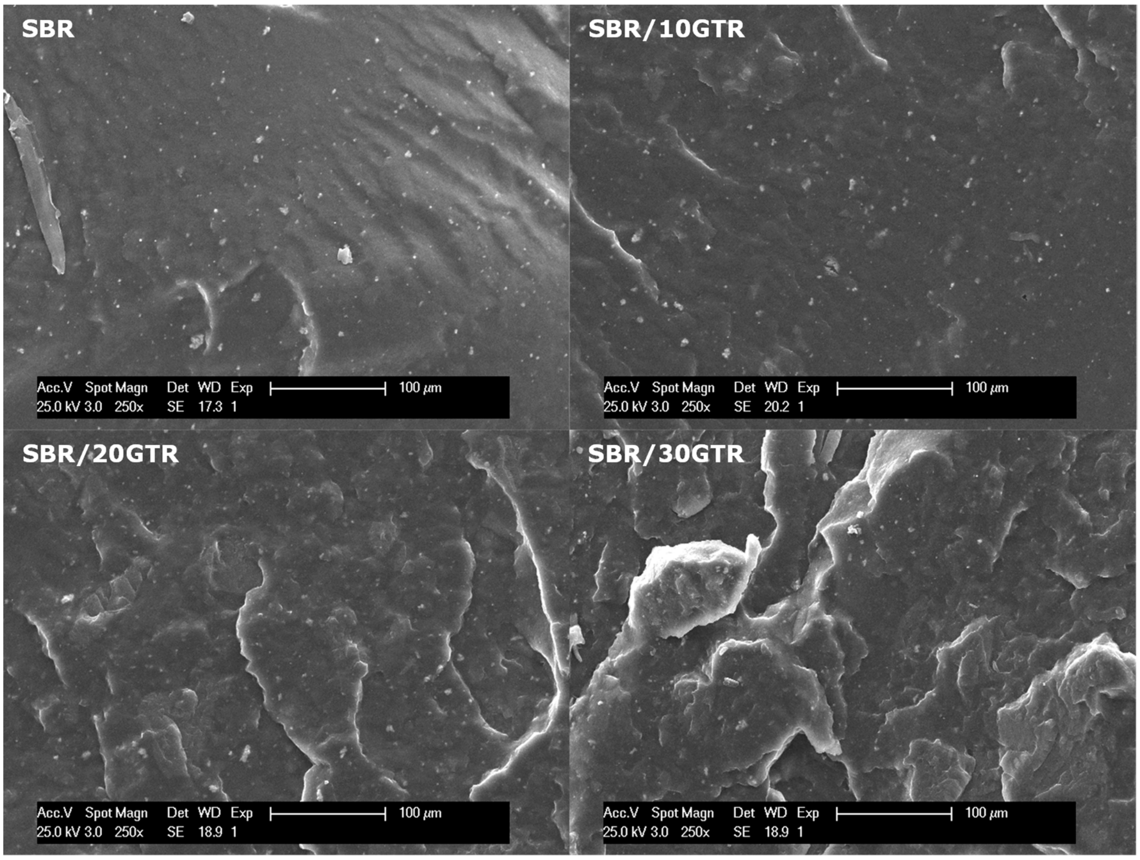

Figure S3-1. Scanning electron microscope (SEM) images of fracture surface of SBR/GTR compounds.

S4. Dielectric properties of SBR/GTR compounds.

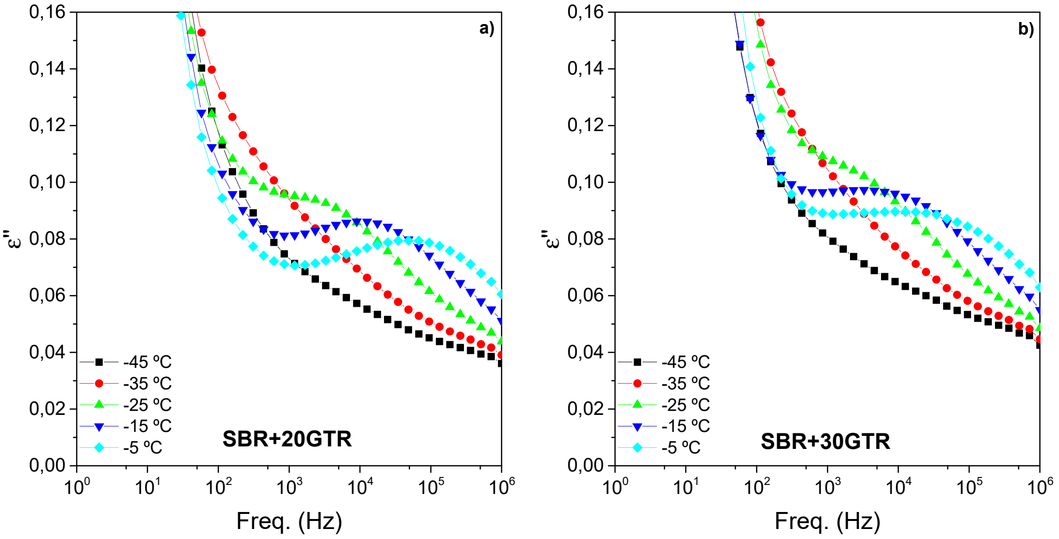

Figure S4-1. Dielectric loss (ε'') as a function of the frequency of: a) SBR/20GTR; b) SBR/30GTR, in the temperature range from -45 to -5 ºC.

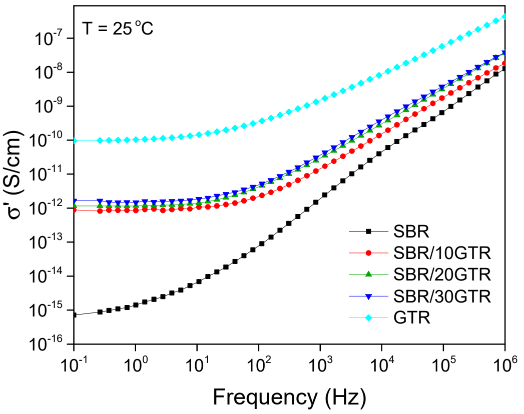

Figure S4-2. Electrical conductivity (σ´) as a function of frequency of SBR/GTR compounds at 25 ºC.
